# Supplementary material for: Gut Microbial Signatures of Broiler Lines Divergently Selected for Inosine Monophosphate and Intramuscular Fat Content
Source: Animals (Basel). 2025 Aug 9;15(16):2337. doi: 10.3390/ani15162337 (PMC12383172; doi:10.3390/ani15162337)
Supplement: Supplementary file 1 [file animals-15-02337-s001.zip › animals-3777907-supplementary.pdf]

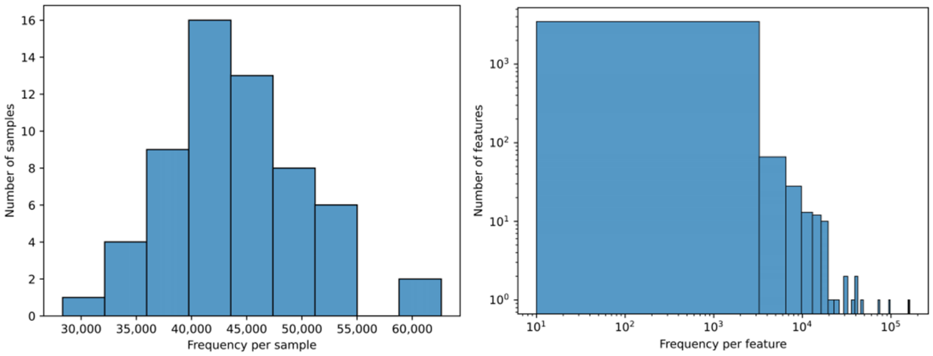


**Figure S1.** Total frequency of each sample and the total frequency of each feature.

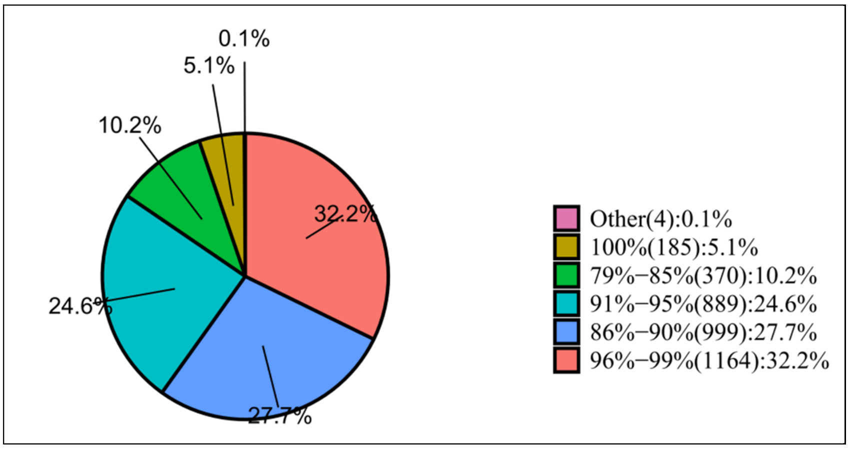


**Figure S2.** BLASTn alignment confidence.

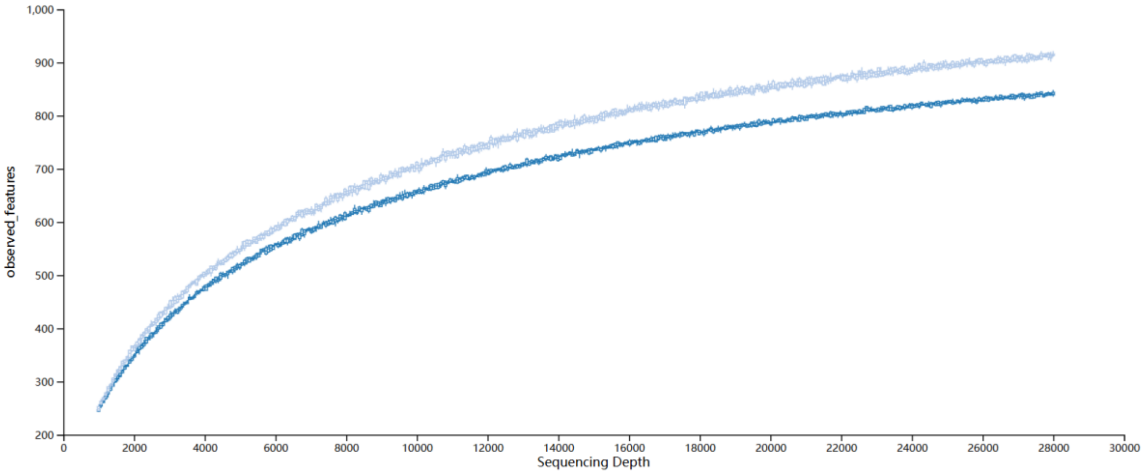


**Figure S3.** Rarefaction curves based on observed ASVs.

.

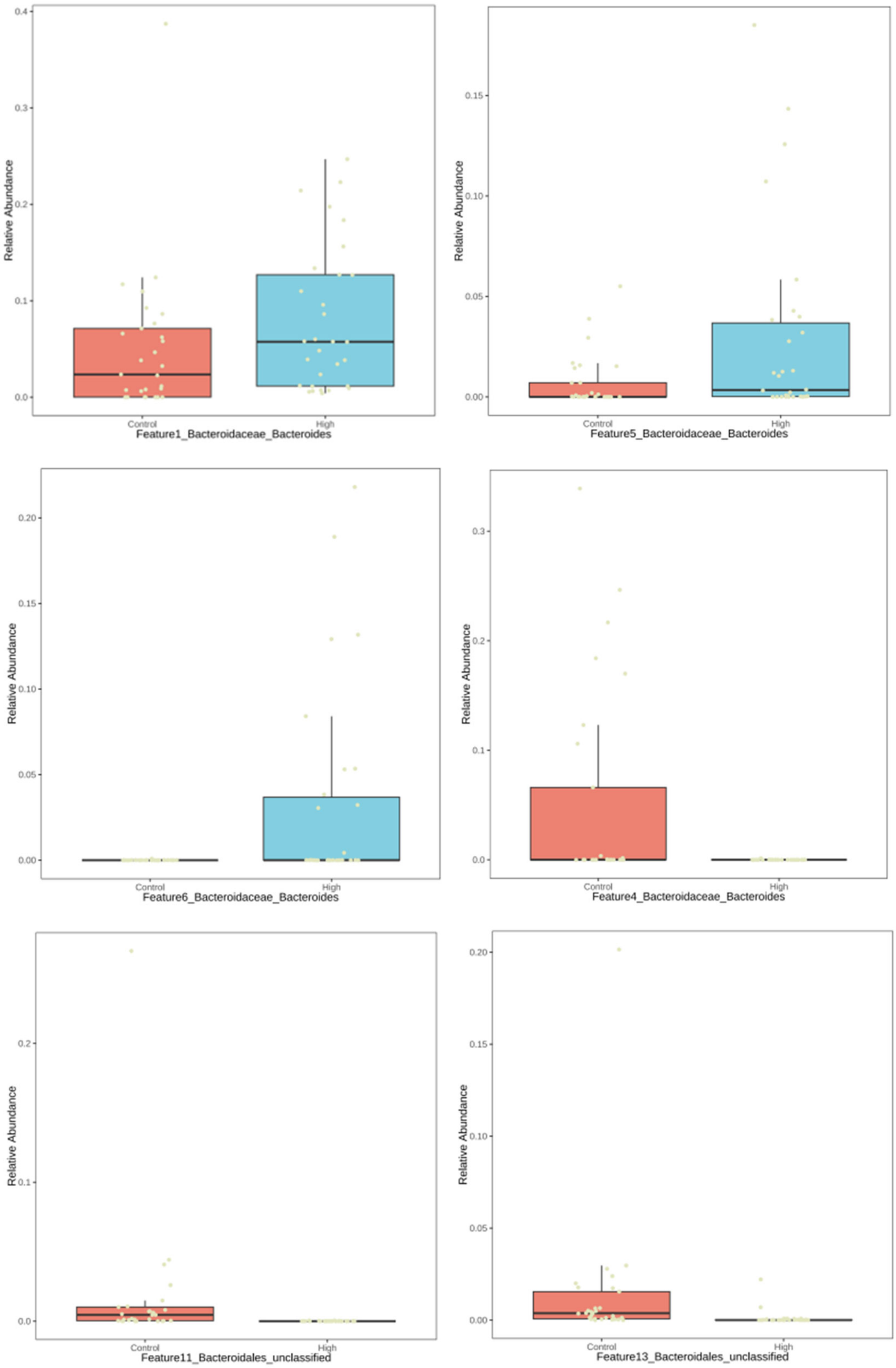


**Figure S4.** Top biomarkers with LDA > 4 (genus Bacteroides).

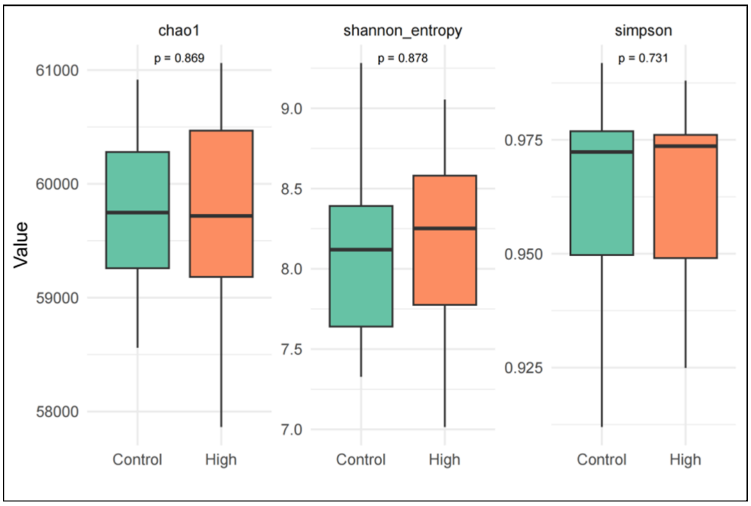


**Figure S5.** Non-significant differences in metagenomic alpha diversity indices between groups.

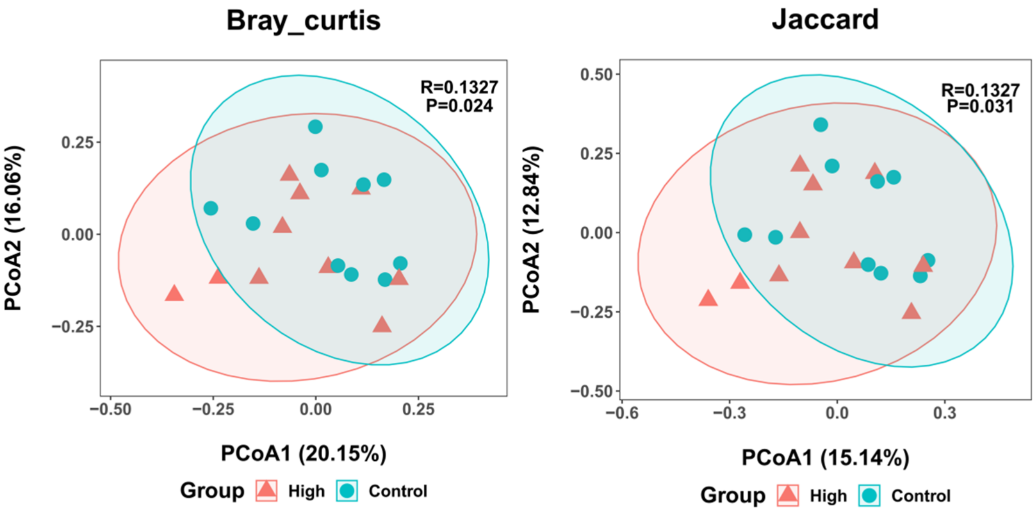


（A）　　　　　　　　（B）

**Figure S6.** Beta diversity analysis of metagenomic data. (A) Bray–Curtis PCoA. (B) Jaccard PCoA.

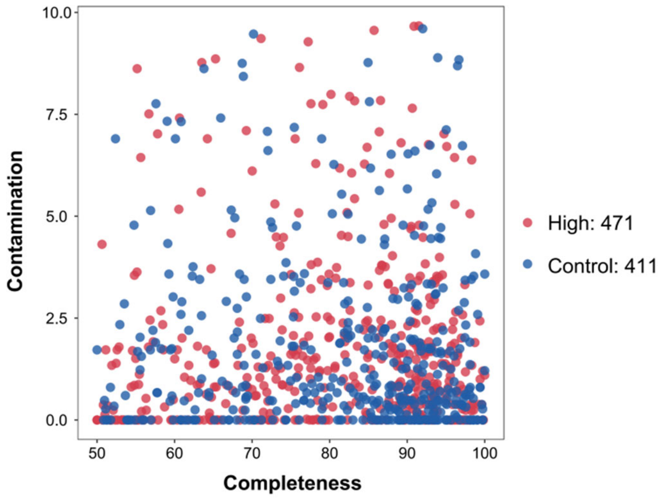


**Figure S7.** Distribution of MAG quality metrics.

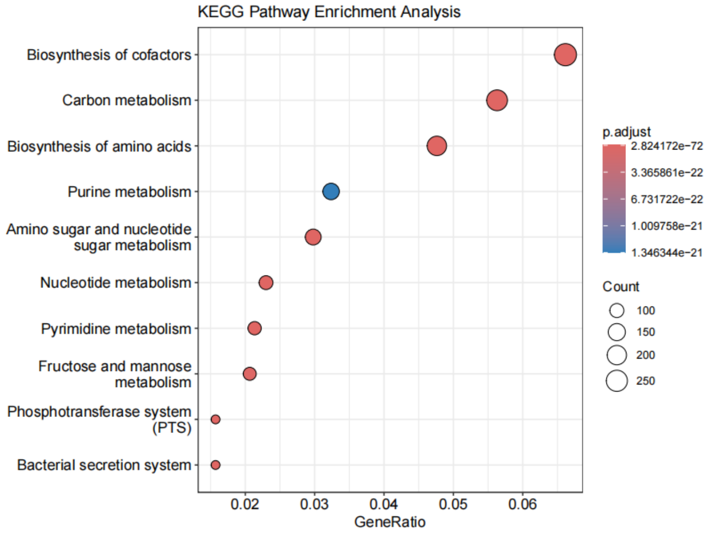


**Figure S8.** Top 10 KEGG pathway enrichment in control group.

**Table S1.** Metagenomic sequencing data quality metrics.

| Sample ID | Raw Reads | Clean Reads | Raw Bases (G) | Clean Bases (G) | Effective (%) | Q20 (%) | Q30 (%) |
|---|---|---|---|---|---|---|---|
| A11 | 67093348 | 66326414 | 10.06 | 9.95 | 98.86 | 97.67 | 93.30 |
| A18 | 71101216 | 70567896 | 10.67 | 10.59 | 99.25 | 97.67 | 93.28 |
| A19 | 66827076 | 66518240 | 10.02 | 9.98 | 99.54 | 98.01 | 94.05 |
| A22 | 67281626 | 66948640 | 10.09 | 10.04 | 99.51 | 98.16 | 94.44 |
| A23 | 69491550 | 69154702 | 10.42 | 10.37 | 99.52 | 98.17 | 94.40 |
| A26 | 68602598 | 68284394 | 10.29 | 10.24 | 99.54 | 97.39 | 92.60 |
| A27 | 66812416 | 66416306 | 10.02 | 9.96 | 99.41 | 98.02 | 93.98 |
| A28 | 69465818 | 69181984 | 10.42 | 10.38 | 99.59 | 97.72 | 93.37 |
| A30 | 68432710 | 68028940 | 10.26 | 10.20 | 99.41 | 98.14 | 94.29 |
| A6 | 67275862 | 66934604 | 10.09 | 10.04 | 99.49 | 97.84 | 93.69 |
| B17 | 68435312 | 68170636 | 10.27 | 10.23 | 99.61 | 97.75 | 93.40 |
| B19 | 65628012 | 65332534 | 9.84 | 9.80 | 99.55 | 98.22 | 94.51 |
| B2 | 75075694 | 74645932 | 11.26 | 11.20 | 99.43 | 98.39 | 94.98 |
| B20 | 78048094 | 77551634 | 11.71 | 11.63 | 99.36 | 98.13 | 94.37 |
| B27 | 68082880 | 67494740 | 10.21 | 10.12 | 99.14 | 97.94 | 93.93 |
| B29 | 68317526 | 67912284 | 10.25 | 10.19 | 99.41 | 98.41 | 95.00 |

| | | | | | | | |
|---|---|---|---|---|---|---|---|
| B3 | 68057292 | 67737940 | 10.21 | 10.16 | 99.53 | 98.24 | 94.58 |
| B5 | 69299368 | 69044458 | 10.39 | 10.36 | 99.63 | 98.05 | 94.11 |
| B6 | 67121440 | 66829716 | 10.07 | 10.02 | 99.57 | 97.86 | 93.68 |
| B8 | 69406200 | 68950300 | 10.41 | 10.34 | 99.34 | 97.90 | 93.81 |

**Table S2.** MAGs statistics for each sample.

| Sample ID | MAGs Count | Average length (bp) | Completion (%) | Contamination (%) | N50 (bp) |
|---|---|---|---|---|---|
| A11 | 58 | 1981254 | 81.21 | 2.00 | 33323 |
| A18 | 31 | 2006833 | 82.34 | 1.90 | 31807 |
| A19 | 52 | 1872764 | 80.38 | 1.72 | 29291 |
| A22 | 50 | 1906403 | 80.02 | 1.66 | 32576 |
| A23 | 52 | 1918063 | 81.36 | 2.14 | 28763 |
| A26 | 53 | 1876080 | 79.58 | 1.35 | 40079 |
| A27 | 52 | 1844734 | 75.84 | 1.88 | 29248 |
| A28 | 35 | 1868841 | 83.79 | 1.23 | 36426 |
| A30 | 40 | 1994295 | 79,00 | 1.89 | 28627 |
| A6 | 48 | 2177127 | 81.95 | 1.12 | 38815 |
| B17 | 37 | 1903364 | 81.07 | 1.60 | 44561 |
| B19 | 40 | 1961480 | 81.32 | 1.31 | 41707 |
| B2 | 52 | 1899700 | 79.38 | 1.91 | 39683 |
| B20 | 28 | 1696862 | 77.48 | 1.60 | 46751 |
| B27 | 41 | 1999516 | 84.42 | 1.87 | 38537 |
| B29 | 16 | 1965368 | 81.67 | 2.17 | 63523 |
| B3 | 42 | 1980987 | 84.93 | 1.47 | 54151 |
| B5 | 48 | 2014227 | 82.27 | 1.93 | 42676 |
| B6 | 46 | 2005733 | 80.84 | 1.93 | 34156 |
| B8 | 61 | 1991212 | 81.54 | 1.62 | 38839 |

**Table S3.** Data sources of 19,628 MAGs.

| Year | Titles | MAGs Count | Data Source URL |
|---|---|---|---|

| | | | |
|---|---|---|---|
| 2021 | Metagenome-Assembled Genomes and Gene Catalog from the Chicken Gut Microbiome Aid in Deciphering Antibiotic Resistomes | 12339 | https://figshare.com/articles/dataset/MAGs/15982089 |
| 2021 | Extensive Microbial Diversity Within the Chicken Gut Microbiome Revealed by Metagenomics and Culture | 5500 | https://figshare.com/articles/dataset/Gene_catalogue_compl_incompl_95_prot_faa_/13116809/4 |
| 2021 | Assembly of Hundreds of Novel Bacterial Genomes from the Chicken Caecum | 469 | https://datashare.ed.ac.uk/handle/10283/3371 |
| 2020 | Newly Explored Fecalibacterium Diversity Is Connected to Age, Lifestyle, Geography, and Disease | 42 | https://data.mendeley.com/datasets/t74rxwrd6z/1 |
| 2021 | Genome-Resolved Metagenomics of the Chicken Gut Microbiome | 155 | https://www.ncbi.nlm.nih.gov/bioproject/?term=PRJNA715658 |
| 2022 | Large Scale Genome-Centric Metagenomic Data from the Gut Microbiome of Food-Producing Animals and Humans | 661 | https://www.ncbi.nlm.nih.gov/nuccore/?term=PRJNA682348 |
| 2019 | Genome Reconstruction of a Novel Carbohydrate Digesting Bacterium from the Chicken Cecal Microflora | 1 | https://www.ncbi.nlm.nih.gov/bioproject/PRJNA435487 |
| 2022 | Improved Microbial Genomes and Gene Catalog of the Chicken Gut from Metagenomic Sequencing of High-Fidelity Long Reads | 461 | https://ftp.cngb.org/pub/gigadb/pub/10.5524/102001_103000/102330/02.microbial_genomes/ |
